# Temperature/Stress-Dependent Fractional Creep Models of Thermoplastic Polymers

**DOI:** 10.3390/polym17141984

**Published:** 2025-07-19

**Authors:** Leixiao Wu, Wei Cai, Jie Yang

**Affiliations:** College of Mechanical and Electrical Engineering, Hohai University, Changzhou 213022, China; 231619010098@hhu.edu.cn (L.W.); 241612010116@hhu.edu.cn (J.Y.)

**Keywords:** fractional model, creep behavior, temperature/stress dependence, Monkman–Grant law

## Abstract

The creep behavior of thermoplastic polymeric materials is highly dependent on loading conditions, which must be accounted for in the intrinsic model. In this paper, fractional creep models have been developed to describe the temperature/stress-dependent creep/creep–recovery and accelerated creep damage behavior, with the construction of a criterion correlating model parameters with temperature and initial stress. The fractional order in the fractional creep/creep–recovery model can be physically interpreted by the well-known master curve, and the creep rupture time can be predicted by combining the Monkman–Grant law with the fractional creep damage model. Extensive experimental data are employed to substantiate the model’s applicability under different loading conditions. Moreover, a comparative analysis highlights the proposed model’s superior simplicity and performance over existing models.

## 1. Introduction

Polymers, which are covalently bonded macromolecules with substantial molecular weights, are extensively utilized in engineering applications due to their low density, high strength, and excellent corrosion resistance [1,2]. Polymer components used in aerospace, automotive, power generation, and polymer systems are exposed to high temperatures and high stress conditions for long periods of time, and the analysis of creep behavior becomes a central issue in design. The researchers have performed rigorous creep experiments on a range of polymer materials, including high-density polyethylene (HDPE) [3,4], polypropylene (PP) [5,6], fiber reinforced plastic [7], and polyethylene terephthalate (PET) [8]. The deformation response of polymers subjected to constant loads at different temperatures indicates the predominant damage mechanism of creep [9]. Thermoplastics also demonstrate significantly diminished creep resistance at elevated temperatures owing to their inherent thermal sensitivity [10,11], which frequently results in complex practical applications [12]. Hence, characterizing creep behavior of engineering materials under varying loading conditions is critical for assessing their long-term reliability and durability.

In recent decades, a substantial number of creep models have been proposed to quantitatively characterize the creep behavior of polymers under constant strain or stress loading. Holzapfel and Gasser [13] proposed a viscoelastic model to describe the creep response of fiber-reinforced composites. Lai and Bakker [14] proposed a unified nonlinear creep criterion to predict the long-term creep response of high-density polyethylene (HDPE), taking into account the effects of physical aging response. Drozdov et al. [15] investigated the observed results of polypropylene/clay nanocomposites in creep tests under various stresses. Medvedev and Caruthers [16] performed a nonlinear creep study on PMMA to generally characterize the deformation response of glassy polymers. Wang et al. [17] conducted experiments on creep and creep–recovery at different temperatures or strain rates in order to understand the creep behavior of different morphologies and employed theoretical models to describe the creep/creep-recovery behavior for a better understanding of the underlying mechanisms. However, the creep or creep–recovery models described above do not take into account the effects of stress and temperature, limiting their extended application.

In order to ensure the long-term durability of PP, creep fracture tests have been conducted at different levels of constant load by Wu et al. [18]. Since the complex long-term behavior of thermoplastic materials is critical for the development of reliable life prediction models, Jorik et al. [19] performed long-term creep measurements on PC at precise temperature conditions to determine their creep rupture time. Nevertheless, the above model does not further discuss the effects of stress and temperature on damage in accelerated creep behavior. In general, the previously referenced ontological models necessitate complex mathematical operations to attain a high degree of accuracy by acquiring multiple material parameters. Consequently, it is advantageous to propose a concise mathematical model with parameters of evident physical significance to characterize the creep behavior of polymers influenced by loading conditions.

Since the pioneering use of fractional operators to characterize constant rate deformations by Smit and Vries [20], fractional derivative models have been developed considerably in the study of the mechanical response for viscoelastic material. Subsequently, the variable-order fractional models have also been constructed to investigate the complex mechanical behaviors of viscoelastic solids and glassy polymers [21,22]. Furthermore, the successful application of fractional models to the simulation of creep responses for various polymers has been universally witnessed [23,24,25]. However, current fractional creep models lack quantitative analysis of loading condition effects, which has restricted their applicability. Meanwhile, the physical interpretation of fractional order in relation to creep rupture time remains unresolved in current modeling frameworks. To address such gap, fractional creep models have been systematically investigated in this manuscript by establishing a quantitative criterion linking model parameters to loading conditions.

The rest of this paper is constructed as follows. Section 2 establishes the fractional creep–recovery and creep damage model under the influence of loading conditions. In Section 3, various experimental data of polymeric materials are applied to verifying the established fractional creep/creep–recovery model, which is further compared with the existing models to demonstrate the applicability and superiority. The developed fractional accelerated creep damage model is successfully employed to characterize experimental data to physically relate the fractional order to rupture time in Section 4. Finally, Section 5 summarizes the main conclusions

## 2. Fractional Creep Model

Nonlinear stress–strain relationships for polymers have been widely observed, thus beyond the conventional descriptions of Hooke’s and Newton’s laws. To address these limitations, various intrinsic models have been proposed to demonstrate a nonlinear stress–strain response, with the aim of better characterizing complex behaviors [26,27]. Among these models, the fractional differential operator model is particularly notable, as it is one of the most widely used to illustrate the transition between linear elasticity and viscosity, which can be expressed as follows [20]:

(1) $$\sigma (t)=E\theta^{\alpha }D^{\alpha }\varepsilon (t),\;0<\alpha <1$$

where *E* denotes the elastic modulus, *θ* represents the relaxation time, *α* is fractional order between 0 and 1, and D*^α^* is the *α*-order fractional derivative operator, defined as [28]

(2) $$D^{\alpha }f(t)=\int_{0}^{t}\frac{{\left(t-\tau \right)}^{-\alpha }}{\Gamma (1-\alpha )}f^{\prime }(\tau )d\tau ,\;0<\alpha <1$$

where Γ (·) denotes the gamma function and *f′*(*τ*) represents first-order derivative.

### 2.1. Temperature/Stress-Dependent Fractional Creep/Creep–Recovery Model

As mentioned above, creep is a typical viscoelastic behavior representing the continuous evolution of deformation with time under constant stress. Therefore, the fractional creep/creep–recovery model has been proposed by Brito-Oliveira et al. [29] and can be expressed as

(3) $$\varepsilon (t)=\left\{\begin{array}{l} \frac{\sigma_{0}}{\varphi_{1}}\frac{t^{\alpha }}{\Gamma \left(1+\alpha \right)},\;t\leq t_{m}\;(\mathrm{creep})\\ \frac{\sigma_{0}}{\Gamma \left(1+\alpha \right)}(\frac{t^{\alpha }}{\varphi_{1}}-\frac{{\left(t-t_{m}\right)}^{\alpha }}{\varphi_{2}}),\;t>t_{m}\;(\mathrm{creep}\;\mathrm{recovery}) \end{array}\right.\;$$

where *t_m_* is the creep–recovery onset time, when *t* ≤ *t_m_* is the creep equation and *t* > *t_m_* is the creep–recovery equation, and *φ*_1_ = *E*_1_*θ*_1_*^α^* and *φ*_2_ = *E*_2_*θ*_2_*^α^* are two independent parameters defined to reduce the parameter uncertainty. It should be pointed out that the fractional value *α* can be obtained by taking logarithm of both sides on the creep equation in Equation (3), which exhibits as the gradient between ln(*ε*) and ln(*t*).

Nevertheless, the model mentioned above does not consider the effect of temperature. As it is generally accepted that increasing temperature enhances relaxation kinetics and consequently diminishes the elastic modulus [30], the relationship between the model parameter *φ*_i_ (*i* = 1, 2) and temperature *T* is established as

(4) $$\ln(\varphi_{i})=-r_{i}T+\lambda_{i},\;i=1,\;2$$

where *r_i_*, and *λ_i_* are constants.

Meanwhile, the initial stress also significantly affects the creep and recovery, so its effect must be taken into account. Considering the complex effect of initial stress on the elastic modulus and relaxation time, the relationship is subsequently constructed as

(5) $$\ln(\varphi_{i})=-u_{i}{\sigma_{0}}^{v_{i}}+q_{i},\;i=1,\;2$$

where *u_i_*, *v_i_* and *q_i_* are constants. In a certain temperature/stress range, the change in fractional order *α* with respect to temperature/stress is insignificant, so the parameter *α* is considered to be independent of temperature/stress, which still exhibits a gradient between ln(*ε*) and ln(*t*). Therefore, the above fractional creep/creep–recovery model can be reformulated as

(6) $$\varepsilon (t,T)=\left\{\begin{array}{l} \frac{\sigma_{0}}{\varphi_{1}(T,\sigma_{0})}\frac{t^{\alpha }}{\Gamma \left(1+\alpha \right)},\;t\leq t_{m}\\ \frac{\sigma_{0}}{\Gamma \left(1+\alpha \right)}(\frac{t^{\alpha }}{\varphi_{1}(T,\sigma_{0})}-\frac{{\left(t-t_{m}\right)}^{\alpha }}{\varphi_{2}(T,\sigma_{0})}),\;t>t_{m} \end{array}\right.$$

### 2.2. Temperature/Stress-Dependent Fractional Accelerated Creep Damage Model

**Figure 1:**
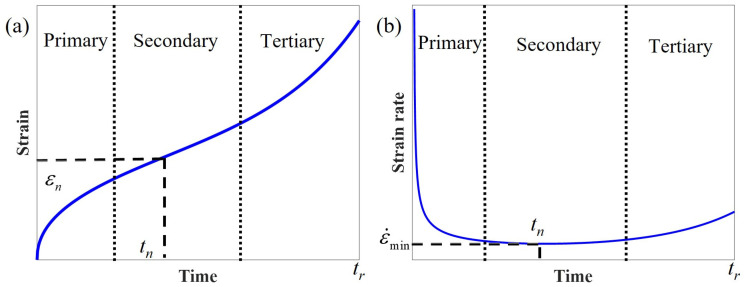
Schematic diagram of creep curves (**a**) accelerated creep, (**b**) creep rate.

As shown in Figure 1a, the entire process of creep deformation can be divided into three stages: primary (transient), secondary (stationary), and tertiary (accelerated) creep. Primary creep is the viscoelastic region, where the strain rate decreases with time. During secondary creep, the strain rate reaches a constant steady plastic flow rate, which gradually accelerates (tertiary creep), eventually leading to failure [31,32].

As the creep process evolves, there will be an accelerated creep phase with a sharp increasing strain, which is usually considered to be seriously damaged. In the reliability analysis, the Weibull distribution function has been widely used to describe the failure process due to its applicability to a variety of complex engineering problems, which is defined as

(7) $$D=1-e^{-at^{b}}$$

where *a* and *b* are the model parameters. In this subsection, based on the damage mechanics theory stress and strain relationship for *σ* = (1 − *D*)*ε* [33], the fractional accelerated creep damage model is established as

(8) $$\varepsilon (t)=\frac{\sigma_{0}t^{\alpha }}{\varphi \left[1-D(t)\right]\Gamma (1+\alpha )},\;\varphi =E\theta^{\alpha }$$

With increasing initial stress, the polymer tends to present a more obvious accelerated creep [34]; therefore, the effect of the initial stress must be taken into account in the constitutive model. In addition, accelerated creep may also be apparently affected by increasing temperature. In particular, the effect of the initial stress/temperature on the damage term *D* and material parameter *φ* is constructed as

(9) $$\left\{\begin{array}{l} a(C)=A_{1}C^{A_{2}}+A_{3}\\ b(C)=B_{1}C^{B_{2}}+B_{3}\\ \varphi (C)=C_{1}C^{C_{2}}+C_{3} \end{array}\right.$$

where *A_i_*, *B_i_*, and *C_i_* (*i* = 1, 2, 3) are constants; *C* represents the loading condition (*σ*_0_ or *T*); and *C*_2_ and *A*_3_ are equal to 0 when *C* stands for *σ*_0_ or *T*. Thus, Equation (8) can be further expressed as

(10) $$\varepsilon (t)=\frac{\sigma_{0}t^{\alpha }}{\varphi (T,\sigma_{0})\left[1-D(t,T,\sigma_{0})\right]\Gamma (1+\alpha )}$$

## 3. Application of Fractional Creep–Recovery Model

In this section, in order to validate the applicability of the proposed model, it is further applied to characterizing the creep/creep-recovery behavior of polymers under different loading conditions.

### 3.1. Modeling Creep/Creep-Recovery Behaviors Affected by Temperature and Initial Stress

Semi-crystalline polypropylene (PP), which has the advantages of low density, ease of processing, recyclability, and excellent mechanical properties, is the second most widely produced synthetic plastic in practical applications demanding long-term durability and reliability. Wang et al. [17] have used a modified injection molding technique using two-shot melt-filling to prepare isotactic polypropylene (IPP) samples with a controllable epidermal core structure, and conducted experiments on creep and recovery at different temperatures (35 °C, 60 °C, and 80 °C) and initial stresses (3 MPa, 4 MPa, and 5 MPa).

**Figure 2:**
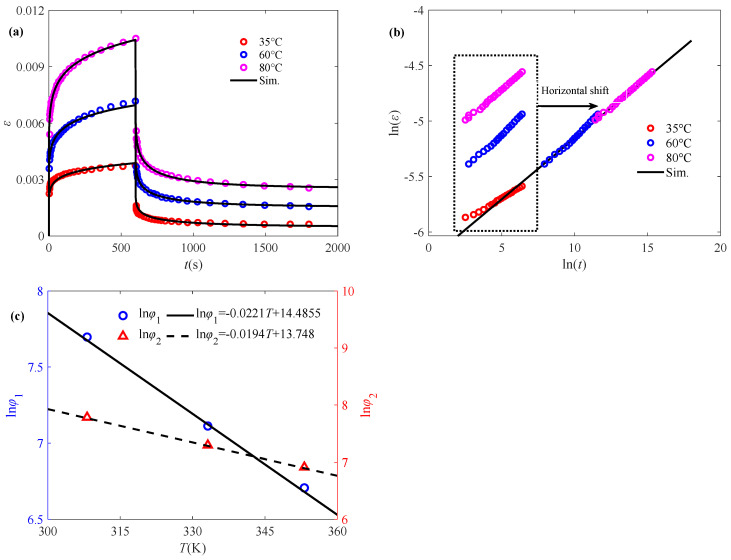
Application of the fractional model in characterizing the creep/creep-recovery behavior of IPP subjected to temperature: (**a**) fitted creep/recovery curves, (**b**) master curve for the creep phase, and (**c**) model parameters ln*φ*_1_ and ln*φ*_2_ versus temperature. The experimental data are quoted from [17].

As illustrated in Figure 2a, it can be seen that the creep and creep-recovery behaviors of IPP tend to nonlinearly increase along with time with a decreasing growth rate, which are apparently affected by temperature. It is evident that a considerable number of researchers have employed the time–temperature superposition principle to articulate the temperature-influenced creep behavior, among which the most prominent one is the time–temperature superposition principle proposed by the Williams–Landel–Ferry equation [35,36]:

(11) $$\mathrm{lg}(a_{T})=-\frac{C_{T1}\left(T-T_{ref}\right)}{C_{T2}+\left(T-T_{ref}\right)}$$

where *C_T_*_1_ and *C_T_*_2_ are constant model parameters reflecting effects of material properties, strain rate and temperature; *T_ref_* denotes the reference temperature; and *a_T_* represents the time–temperature displacement factor. In this subsection, the proposed model, Equation (6), is employed to characterize such phenomenon. The corresponding master curves can be obtained by superimposing the horizontal displacement curves corresponding to different ambient temperatures on the logarithmic time scale. The logarithmic relationship of strain versus time in the creep stage obtained through Equation (11) shows a linear trend, as shown in Figure 2b. Therefore, the slope of the master curve corresponds to a fractional order *α* value of 0.11 at different temperatures. In addition, the parameter *φ*_1_ can be further determined according to Equation (6). It is worth noting that a constant *α* leads to the constant material parameter *φ*_1_ during creep at a specific temperature, which is in good agreement with the results obtained from existing polymer rheology experiments [37]. The change in temperature leads to the degradation of the elastic modulus, and accelerates the relaxation process, causing a smaller ln(*φ*_1_). As seen in Figure 2c, ln(*φ*_1_) satisfies a linearly decreasing relationship of the temperature *T*, suggesting that such parameter can capture the property evolution affected by temperature.

Numerical simulation reveals that the fractional order at the creep–recovery stage remains the same as that at the creep stage. Hence, the value of parameter *φ*_2_ at different temperatures can be determined on the basis of Equation (6), which presents that ln*φ*_2_ also exhibits a linear temperature-dependent decline, consistent with Equation (4), as shown in Figure 2c. It can be observed from Figure 2a that the creep/creep–recovery response described by the proposed temperature-dependent model is very close to the experimental data, which manifests that the established fractional model is an effective tool to characterize the temperature affected creep behaviors of IPP.

**Figure 3:**
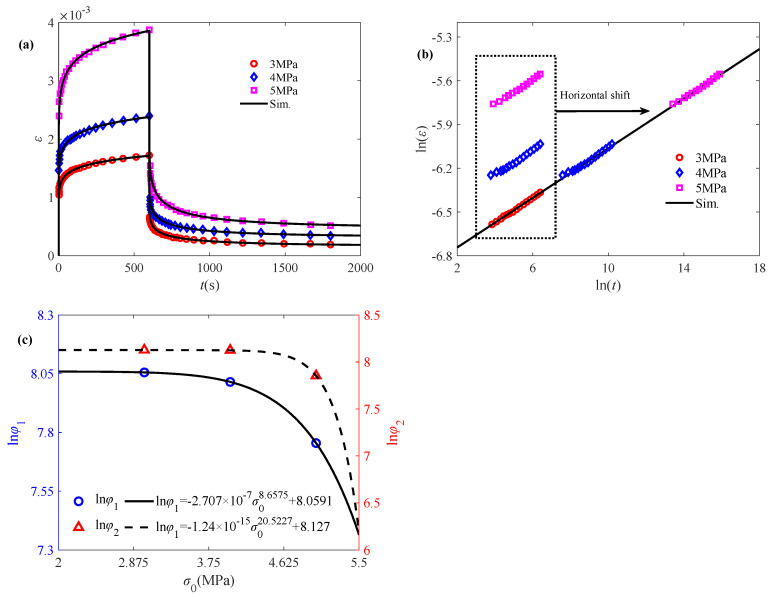
Application of the fractional model in characterizing the creep/creep-recovery behavior of IPP subjected to initial stress: (**a**) fitted creep/recovery curves, (**b**) master curve for the creep phase, and (**c**) model parameters ln*φ*_1_ and ln*φ*_2_ versus initial stress. The experimental data are quoted from [17].

As demonstrated in Figure 3a, the initial stress also exerts a substantial influence on the creep/creep-recovery behavior, i.e., increasing initial stress results in an enhancement of creep strain. Analogous to the time–temperature superposition principle, the time–stress superposition principle is also employed to construct the master curve for creep behavior, which is defined as [38]

(12) $$\mathrm{lg}(\phi_{\sigma })=-\frac{C_{\sigma 1}(\sigma -\sigma_{ref})}{C_{\sigma 2}+(\sigma -\sigma_{ref})}$$

where *C_σ_*_1_ and *C_σ_*_2_ are constant model parameters reflecting material/loading-dependent properties, *σ_ref_* represents the reference stress, and *ϕ*_σ_ denotes the stress transfer factor.

The established time–stress master curve obtained through Equation (12) is shown in Figure 3b, and the value of the fractional order agrees with the slope of the stress master curve as 0.085. The model parameters ln(*φ*_1_) and ln(*φ*_2_) can be subsequently obtained through Equation (6), as shown in Figure 3c, which are in a power law decreasing relationship, indicating the more complex effect of initial stress on the elastic modulus. It can also be found from Figure 3a that the proposed model is able to well characterize the creep/creep-recovery behavior affected by the initial stress.

### 3.2. Validation of the Proposed Model

To further demonstrate the feasibility in characterizing the creep/creep-recovery behavior of polymeric materials subjected to temperature or initial stress, an additional set of experimental data will be used for validation.

**Figure 4:**
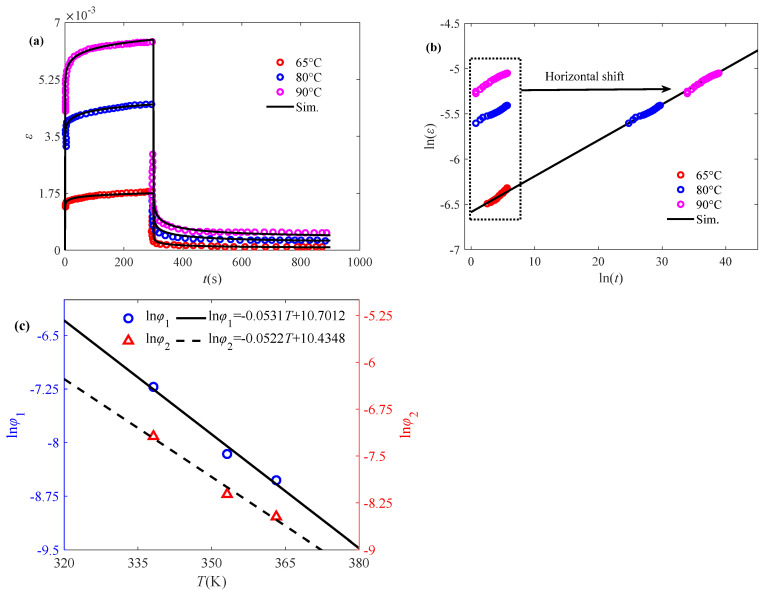
Validation of the proposed fractional model in characterizing the creep/creep-recovery behavior of emulsion-filled gels subjected to temperature: (**a**) fitted creep/recovery curves, (**b**) master curve for the creep phase, and (**c**) model parameters lnφ_1_ and ln*φ*_2_ versus temperature. The experimental data are quoted from [39].

Temperature exerts a substantial influence on the structural design of starch-based emulsion-filled gels, with rheology as the preferred technique for quantifying the properties of emulsion-filled gels. Consequently, Zhao et al. [39] investigate the role of gelation temperature in the formation of emulsion-filled gels with starch as emulsifier and polymer gel matrix through creep/creep–recovery experiments. Three sets of experimental data at 65 °C, 80 °C, and 90 °C are selected for the purpose of validation. The temperature master curve shown in Figure 4b can still be characterized using a linear function. As demonstrated in Figure 4c, the model parameter ln(*φ*_1_) and ln(*φ*_2_) still satisfy the linear relationship with respect to temperature *T*. The creep/creep-recovery behavior of the emulsion-filled gel is described by Figure 4a, which shows that the proposed intrinsic model is in good agreement with the experimental data, thus confirming the rationality of the proposed model.

**Figure 5:**
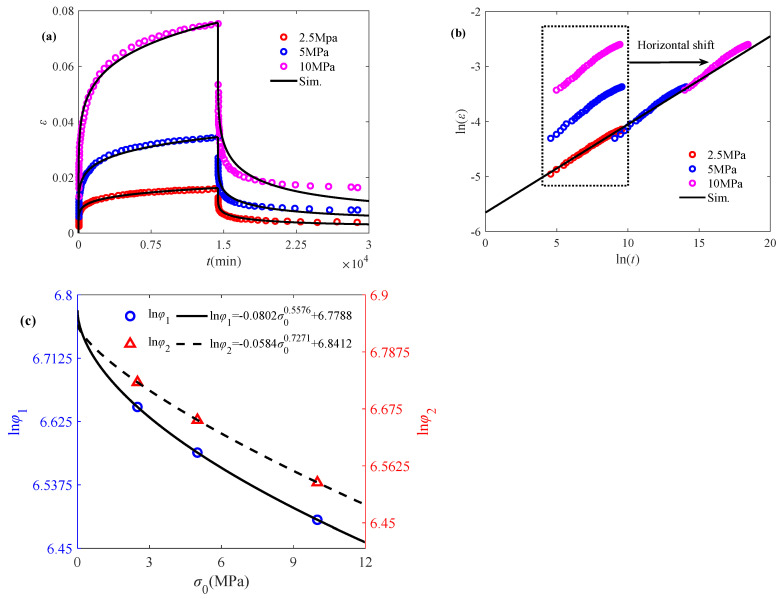
Validation of the fractional model in characterizing the creep/creep-recovery behavior of UHMWPE subjected to initial stress: (**a**) fitted creep/recovery curves, (**b**) master curve for the creep phase, and (**c**) model parameters ln*φ*_1_ and ln*φ*_2_ versus initial stress. The experimental data are quoted from [40].

Ultra-high molecular weight polyethylene (UHMWPE) is increasingly utilized in industrial applications due to its wear and impact resistance. As a viscoelastic material, it exhibits inherent weaknesses, including creep resistance and fatigue strength. Another set of experimental data of UHMWPE-GUR 410 medical-grade material under different loading conditions (2.5 MPa, 5 MPa, and 10 MPa), shown in Figure 5a, is employed to validate the feasibility of the proposed model [40].

From Figure 5b, it can be seen that the experimental data under different stresses have a linear relationship in the double logarithmic plot, while the magnitude of the slope of the main curve agrees with the magnitude of the fractional order as 0.1602. From Figure 5a, it can be found that the creep/recovery behaviors of UHMWPE become more significant under the initial stress of 10 MPa, which further suggests that the change in the initial stress affects the creep/recovery more significantly, and thus compared to the relationship with the temperature changes, the model parameters ln(*φ*_1_) and ln(*φ*_2_) show a power-law relationship with respect to the initial stress, as shown in Figure 5c. It is well demonstrated by Figure 5a that the creep/creep-recovery behavior of UHMWPE is well characterized by the proposed model.

In summary, the fractional creep/creep-recovery model is capable of adequately describing the temperature/stress-dependent creep/creep-recovery behavior, with the model parameter ln(*φ_i_*) defined as a linear function of temperature or a power-law function of applied stress.

### 3.3. Model Comparison

To demonstrate the superiority of the proposed fractional creep model, the fractional creep/creep–recovery model is compared with the existing model. In order to quantitatively evaluate the imitative effects of the models, the fitting accuracy is evaluated by using *R*^2^, which is defined as

(13) $$R^{2}=1-\frac{\sum {\left(Y_{\mathrm{actual}}-Y_{\mathrm{predict}}\right)}^{2}}{\sum {\left(Y_{\mathrm{actual}}-Y_{\mathrm{mean}}\right)}^{2}}$$

where *Y*_actual_ and *Y*_predict_ represent experimental data and predicted value, respectively, and *Y*_mean_ is the mean value.

The Findley power-law creep model is a well-known mathematical model used to describe the creep behavior of materials at constant stress and temperature. The model was proposed by Findley, and primarily used to predict the change in strain with time for a material under long-term stress, of which the creep formulation can be expressed as follows [41]:

(14) $$\varepsilon (t)=a_{0}+dt^{w}$$

where *a*_0_, *d*_0_, and *w* are model parameters. It has been further modified for demonstrating creep–recovery as [42]

(15) $$\varepsilon (t)=dt^{w}-d_{1}{\left(t-t_{m}\right)}^{w_{1}}$$

where *d*_1_ and *w*_1_ are model parameters, respectively, and *t_m_* is the creep–recovery onset time. The Findley model is an empirical model and can be regarded as a simplified form of the nonlinear viscoelastic model, applicable to polymer, soft and metallic materials, without considering temperature and stress dependence.

**Figure 6:**
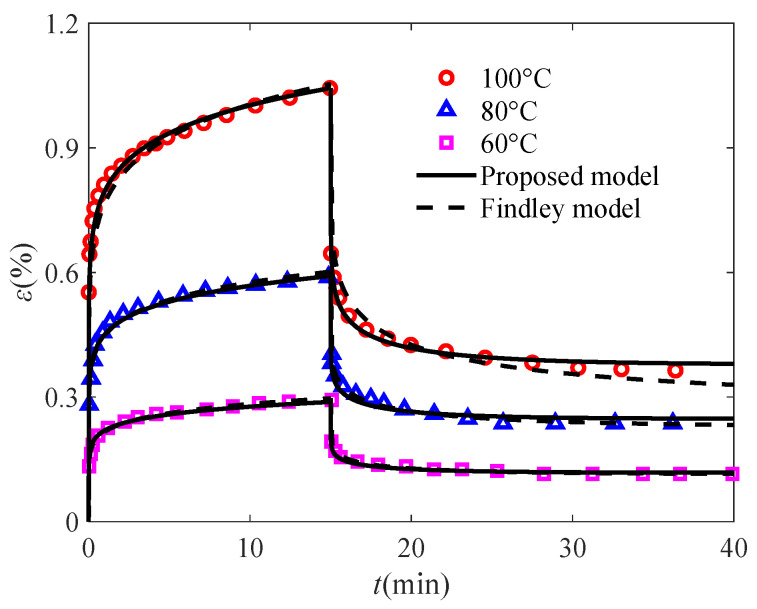
Comparison between fractional creep/creep–recovery models and the Findley model (the experimental data are quoted from [43]).

As can be seen from Figure 6 and Table 1, the *R*^2^ values of both models for describing the UHMWPE/CNTs composite fibers at 100 °C, 80 °C, and 60 °C are greater than 0.9, but the proposed model has a higher fitting accuracy, which further confirms the superiority of the proposed model and suggests that it is an effective tool to capture the creep/creep–recovery of the polymers. In addition, the modified Findley power-law model proposed by Mei et al. [42] does not take into account the effects of temperature and stress, thus demanding sixteen parameters to characterize such behavior at three different temperatures, whereas the proposed model requires only seven parameters.

## 4. Fractional Accelerated Creep Damage Model

The accelerated creep phase is characterized by the onset of material damage, which induces progressively accelerating deformation until structural fracture occurs. Consequently, it is imperative to predict the creep fracture time, which can be well predicted by the well-known Monkman–Grant law, defined as [44,45,46,47]

(16) $$C_{MG}=t_{r}{\left({\dot{\varepsilon }}_{\min}\right)}^{v}$$

where *C_MG_* is the Monkman–Grant parameter, *t_r_* denotes the creep fracture time, $\dot{\varepsilon }$_min_ stands for the minimum creep rate, and *v* is a material constant. As discussed above, in the secondary creep phase, the creep rate undergoes a gradual change and displays a minimum value, as illustrated in Figure 1b. It is evident that a specific relationship exists between such minimum value and the creep rupture time. As long as such two parameters can be linearly represented in a double logarithmic plot, the Monkman–Grant law holds. According to Equation (8), the creep rate combined with the fractional creep damage model can be derived as

(17) $$\dot{\varepsilon }(t)=\frac{e^{at^{b}}\sigma_{0}t^{\alpha -1}(abt^{b}+\alpha )}{\varphi \Gamma (1+\alpha )}$$

### 4.1. Application of Accelerated Creep Damage Model

Evaluating the service life of composite structures is one of the key issues in their design, and creep damage experiments are an effective method of assessing long-term strength under time-independent loading. Drozdov et al. [15] investigate the observed results of polypropylene/clay nanocomposites in creep tests at a variety of stresses, where the experimental data of NC1 (nano clay concentration of 1 wt.%) at different initial stresses (18.5 MPa, 19 MPa, 20 MPa, and 21 MPa) are selected for analysis.

**Figure 7:**
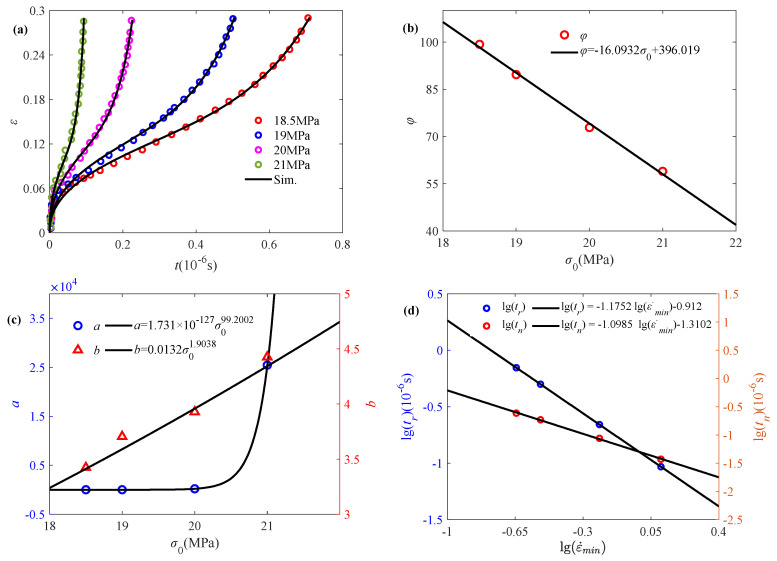
Application of the fractional model in characterizing the accelerated creep behavior of NC1 affected by initial stress: (**a**) simulation of accelerated creep experimental data, (**b**) model parameter *φ* versus initial stress, (**c**) damage parameters *a* and *b* versus initial stress, (**d**) fitting results based on the Monkman–Grant law (the experimental data are quoted from [15]).

Figure 7a reveals that the accelerated creep phenomenon is more obvious with the increase in stress. The fractional order *α* can be obtained as a fixed value of 0.443, according to Equation (8), while the obtained model parameter *φ* is linearly decreasing with the initial stress, as shown in Figure 7b. With the accelerated creep, the degree of damage to the material is increasing. It can be subsequently characterized by the Weibull distribution function, with the parameters *a* and *b* exhibiting a power-law increasing relationship with respect to temperature, as shown in Figure 7c. It indicates that the damage coefficient changes significantly with increasing stress, and a large number of cracks appear within the material. As shown in Figure 7d, creep rupture time *t_r_* and minimum creep rate time *t_n_* apparently linearly depend on minimum creep rate $\dot{\varepsilon }$_min_ in double logarithmic coordinate, where the minimum creep rate time and minimum creep rate can be obtained by Equation (17) and the creep rupture time is obtained from experimental data. It is apparent that the combination of Monkman–Grant law and fractional creep damage model well characterize the creep rupture time of polymers.

**Figure 8:**
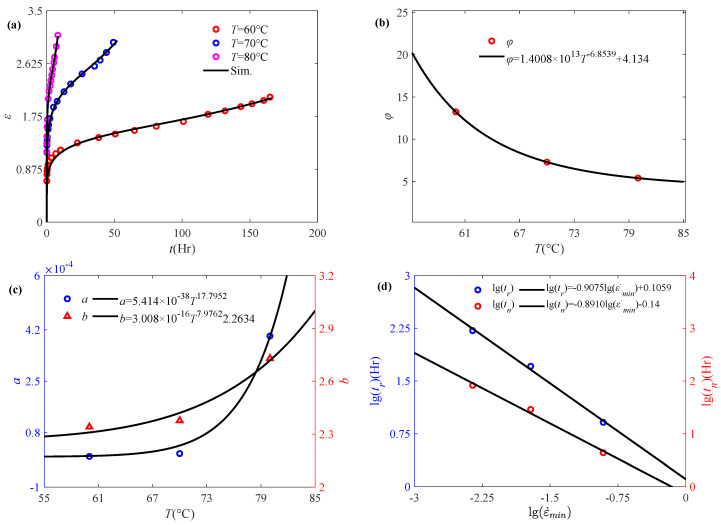
Application of the fractional model in characterizing the accelerated creep behavior of polypropylene composites subjected to temperature: (**a**) accelerated creep curves, (**b**) model parameters *φ* vs. temperature, (**c**) parameters *a* and *b* vs. temperature, (**d**) fitting effects based on the Monkman–Grant law (the experimental data are quoted from [48]).

Nuñez et al. [48] have conducted short- and long-term creep tests on composites prepared from wood flour and polypropylene at different temperatures, where long term creep data at 60 °C, 70 °C and 80 °C are selected for analysis. From Figure 8a, it can be found that the strain response of polypropylene composites during creep increases with temperature, which indicates a significant thermal influence on creep behavior. Therefore, the proposed fractional accelerated damage creep model is used to describe the strain-time curves at different temperatures, and the fractional order *α* is obtained accordingly as 0.15. The model parameter *φ* has a power law decreasing relationship with temperature as shown in Figure 8b, suggesting a more complex thermal effect compared with the stress effect. Figure 8c reveals that parameters (*a* and *b*) in the damage function exhibit a power law dependence on temperature. Elevated temperature, like higher initial stress, induces faster changes in the damage coefficient. Figure 8d shows that both the creep rupture time *t_r_* and minimum creep rate time *t_n_* exhibit a linear decreasing relationship with minimum creep rate $\dot{\varepsilon }$_min_ in double logarithmic coordinate. It indicates that the minimum creep rate and creep rupture time obtained by the proposed temperature-dependent fractional accelerated creep damage model continue to satisfy the Monkman–Grant law.

### 4.2. Validation of the Proposed Model

To further illustrate the feasibility of the proposed model in characterizing the accelerated creep behavior of polymeric materials under different temperatures or initial stresses, it will be validated using an additional set of experimental data, respectively.

**Figure 9:**
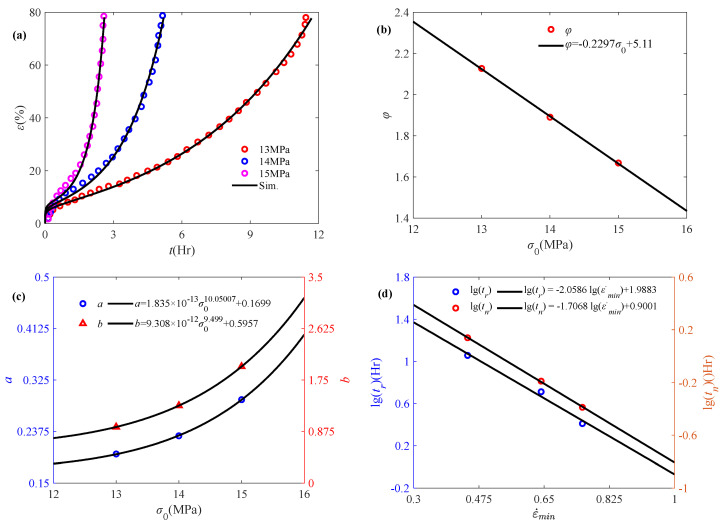
Validation of the fractional model in characterizing the accelerated creep behavior of HDPE subject to initial stress: (**a**) accelerated creep curve, (**b**) model parameter *φ* versus initial stress, (**c**) parameters *a* and *b* versus initial stress, (**d**) fitting effects based on the Monkman–Grant law (the experimental data are quoted from [47]).

Amjadi and Fatemi [47] have conducted a nonlinear creep study on HDPE, among which three sets of experimental data (13 MPa, 14 MPa, and 15 MPa) are selected for analysis, as shown in Figure 9a. The fractional order *α* is obtained as a fixed value of 0.165. With the help of Equation (8), the pattern of parameters *φ*, *a*, and *b*, with respect to initial stress, is still maintained. In addition, the relationship between creep rupture time and minimum creep rate of HDPE still satisfies the Monkman–Grant law, as shown in Figure 9d.

**Figure 10:**
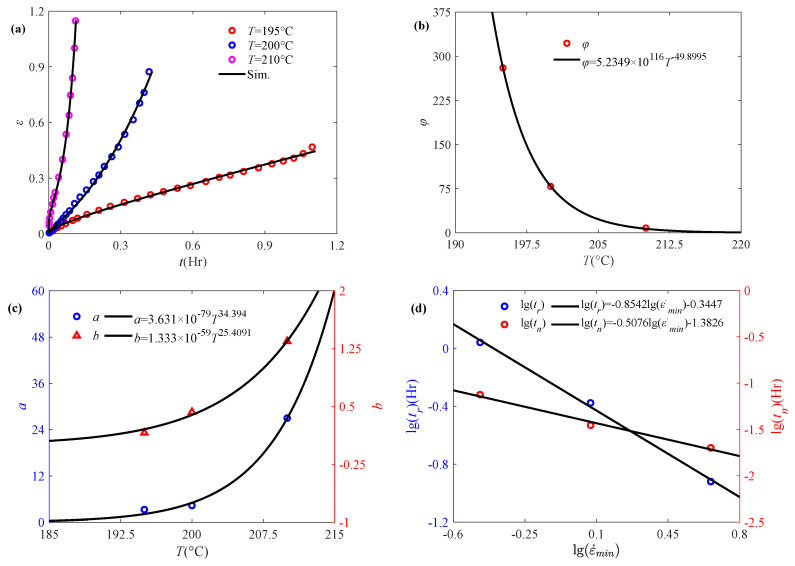
Validation of the fractional model in characterizing the accelerated creep behavior of SCF/PEI composites subjected to temperature (**a**) accelerated creep curves, (**b**) model parameter *φ* vs. temperature, (**c**) parameters *a* and b vs. temperature, (**d**) effect of fitting based on the Monkman–Grant law (The experimental data are quoted from [49]).

Zhang et al. [49] investigated the tensile creep behavior of short carbon fiber reinforced polyetherimide (SCF/PEI) composites at different ambient temperatures, and the effect of fiber surface treatment on the creep behavior was discussed. Among the test data at different temperatures, 195 °C, 200 °C, and 210 °C are selected for model validation due to the significant accelerated creep phenomenon occurred at too high temperature, as shown in Figure 10a.

In order to verify the applicability of the proposed temperature-dependent accelerated creep damage model, the experimental data at different temperatures are selected based on the 4 MPa, and the model parameters are obtained based on the above discussion. Specially, i.e., the fractional order *α* is achieved as 0.32, and the parameters *φ*, *a* and *b* can be accordingly obtained and shown in Figure 10b,c, which still satisfy the power-law relationship with respect to temperature. The relationship between creep rupture time and minimum creep rate as shown in Figure 10d still satisfies the Monkman–Grant law. It can be seen from Figure 10a that the proposed model is able to characterize the temperature affected accelerated creep. The results show that the temperature-dependent fractional accelerated creep damage model is able to describe the complete creep deformation stage, indicating the applicability of the proposed model.

**Figure 11:**
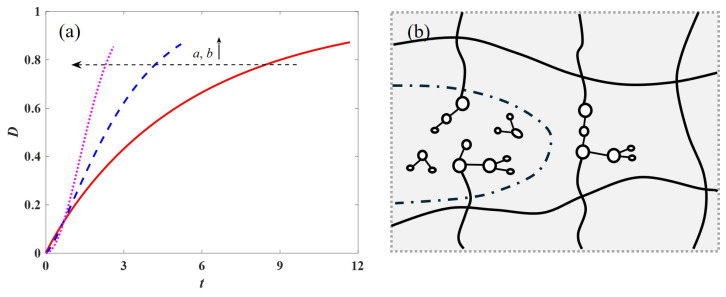
Damage function and microstructure: (**a**) effect of parameters *a* and *b* on the damage function; (**b**) schematic representation of the influence of breaking molecular chains on the widened cracks.

Moreover, the power law dependence of parameters *a* and *b* on stress and temperature in the Weibull damage function captured by Equation (9) clearly demonstrates that increasing stress and temperature reduces the polymer material’s resistance to deformation. Under stress, micron/nano-scale cracks sprout inside the material or at grain boundaries. Crack extension follows fracture mechanics, leading to macroscopic strain accumulation. At high temperatures, the crack tip may undergo oxidation or diffusion, accelerating the expansion. As shown in Figure 11, increasing damage factors *a* and *b* accelerates the damage coefficient *D*, further promotes bond breakage and chain scission, and ultimately leads to creep fracture of the polymeric material [50]. In summary, the accelerated creep behavior of polymers can be well captured by the proposed initial stress- or temperature-dependent fractional accelerated creep damage model, which demonstrates the applicability of the proposed model.

### 4.3. Model Comparison

Given that crack damage in polymers manifests across multiple scales, its underlying mechanisms can be elucidated by examining damage morphology and evolution at both microscopic and macroscopic levels. Li et al. [34] conducted relevant experiments on PMMA and for the first time established a framework for the fracture evolution of creep in glass polymers, which was validated by fractal theory and expressed as follows

(18) $$\sigma +\frac{\eta }{K_{1}+K_{2}}\dot{\sigma }-\frac{\eta }{K_{1}+K_{2}}\frac{2\sigma }{1+\varepsilon }\dot{\varepsilon }=\frac{K_{1}K_{2}}{K_{1}+K_{2}}\left(\varepsilon +\frac{\varepsilon^{2}}{2}\right){\left(1+\varepsilon \right)}^{2}+\frac{K_{1}\eta }{K_{1}+K_{2}}{\left(1+\varepsilon \right)}^{3}\dot{\varepsilon }$$

where *σ* and $\dot{\sigma }$ are the Cauchy stress and its rate, *ε* and $\dot{\varepsilon }$ represent the total strain and total strain rate, respectively, *η* is the viscosity of the dashpot, and *K*_1_ and *K*_2_ denote the elastic modulus.

**Figure 12:**
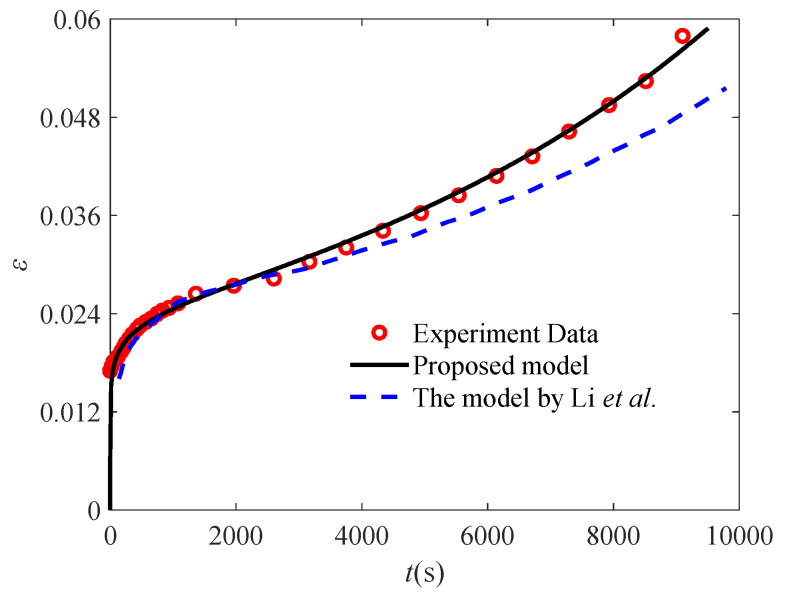
Comparison of fractional accelerated damage creep model with the model by Li et al. (the experimental data are quoted from [34]).

To elucidate the superiority the proposed fractional accelerated creep model, it is further compared with the above-mentioned model on the basis of the experimental data, as shown in Figure 12. It is clear from Figure 12 that the proposed fractional model is closer to the experimental data. Meanwhile, the model proposed by Li et al. is more complex, while the proposed model is more concise. Table 2 shows the relevant parameter values of the proposed model. In summary, the mathematical form of the proposed model is simpler and can achieve higher fitting accuracy than the existing model described above.

## 5. Conclusions

In this paper, the fractional creep model is proposed to characterize the creep behavior and quantify the effects of loading stress and ambient temperature, where the fractional order ranges from 0 to 1, representing an intermediate state between pure elasticity and viscosity. Various creep/creep–recovery and accelerated creep experimental data are used to evaluate the rationality and validity of the proposed model. The main conclusions are summarized below.

By applying a logarithmic transformation to the experimental creep data, the fractional order in the creep stage at different temperatures or initial stresses can be obtained directly, and it is in good agreement with the master curve, which clearly explains the physical significance of the fractional order. The fractional order in the creep–recovery stage remains the same. The parameter ln(*φ_i_*) shows a linear and power law relationship with temperature and initial stress, respectively. The proposed model can characterize the creep/creep-recovery behavior of polymer materials under different stress and temperature conditions with high accuracy.

Therefore, to account for the effect of stress/temperature on damage, the damage coefficient based on the Weibull distribution function are established to characterize the accumulated creep damage, with the construction of the power law dependence of parameters *a*, *b* on stress or temperature. It is found that both stress and temperature significantly influence the damage coefficients. Meanwhile, the model parameter *φ* is linearly and power law decreasing with stress and temperature, respectively, suggesting that increasing either stress or temperature decreases the material parameter *φ*, but the thermal effect is more complex. For the creep rupture time dominated by the accelerated creep phase, it can be predicted by the combination of Monkman–Grant law and fractional accelerated creep damage model. The creep rupture time and minimum creep rate time linearly relates to the minimum creep rate. Compared with the existing model, the proposed intrinsic model possesses simpler formulation, clear physical interpretation, and higher fitting accuracy.

In conclusion, under limited loading conditions, the established temperature- or initial stress-dependent fractional creep/recovery model and fractional accelerated creep damage model can be considered as effective tools for describing the creep behavior of polymeric materials. Future work will further explore the quantitative relationship between fractional order and material microstructure (e.g., molecular chain entanglement density, porosity) and combine fractional order modeling with machine learning to invert fractional order and material constants from limited data.

## Figures and Tables

**Table 1:** The fitting accuracy of the two models.

	*T* (°C)	100 °C	80 °C	60 °C
*R* ^2^				
Proposed model		0.9842	0.9773	0.9854
Findley model		0.9127	0.9750	0.9810

**Table 2:** Parameters of the fractional accelerated damage creep model.

*σ*_0_ (MPa)	*α*	*φ*	*a*	*b*
41.67	0.11	3917.8	9.3428e-7	1.4687

## Data Availability

All relevant data are within the paper.

## References

[1] Charitidis C., Sebastiani M., Goldbeck G. (2022). Fostering research and innovation in materials manufacturing for industry 5.0: The key role of domain intertwining between materials characterization, modelling and data science. Mater. Design.

[2] Lazaridou A., Duta D., Papageorgiou M., Belc N., Biliaderis C.G. (2007). Effects of hydrocolloids on dough rheology and bread quality parameters in gluten-free formulations. J. Food Eng..

[3] Siengchin S., Rungsardthong V. (2013). Hdpe reinforced with nanoparticle, natural and animal fibers: Morphology, thermal, mechanical, stress relaxation, water absorption and impact properties. J. Thermoplast. Compos..

[4] Xu Y.J., Wu Q.L., Lei Y., Yao F. (2010). Creep behavior of bagasse fiber reinforced polymer composites. Bioresource technol..

[5] Siengchin S., Dangtungee R. (2014). Polyethylene and polypropylene hybrid composites based on nano silicon dioxide and different flax structures. J. Thermoplast. Compos..

[6] Pedrazzoli D., Pegoretti A. (2014). Long-term creep behavior of polypropylene/fumed silica nanocomposites estimated by time-temperature and time-strain superposition approaches. Polym. Bull..

[7] Mishnev M., Korolev A., Zadorin A. (2024). Effect of thermal aging on viscoelastic behavior of thermosetting polymers under mechanical and cyclic temperature impact. Polymers.

[8] Qiu J.F., Ma S.Q., Wang S., Tang Z.B., Li Q., Tian A.P., Xu X.W., Wang B.B., Lu N., Zhu J. (2021). Upcycling of polyethylene terephthalate to continuously reprocessable vitrimers through reactive extrusion. Macromolecules.

[9] Gao Y.F., Zhao B., Yin D.S., Yuan L.L. (2022). A general fractional model of creep response for polymer materials: Simulation and model comparison. J. Appl. Polym. Sci..

[10] Grelle T., Wolff D., Jaunich M. (2015). Temperature-dependent leak tightness of elastomer seals after partial and rapid. release of compression. Polym. Test..

[11] Grelle T., Wolff D., Jaunich M. (2017). Leakage behaviour of elastomer seals under dynamic unloading conditions at low temperatures. Polym. Test..

[12] Morra P.V., Radelaar S., Yandouzi M., Chen J., Bottger A.J. (2009). Precipitate coarsening-induced plasticity: Low temperature creep behaviour of tempered sae 52100. Int. J. Plasticity.

[13] Holzapfel G.A., Gasser T.C. (2001). A viscoelastic model for fiber-reinforced composites at finite strains: Continuum basis, computational aspects and applications. Comput. Methods Appl. Mech. Eng..

[14] Lai J., Bakker A. (1995). Analysis of the non-linear creep of high-density polyethylene. Polymer.

[15] Drozdov A., Lejre A.-L.H., Christiansen J.D. (2009). Viscoelasticity, viscoplasticity, and creep failure of polypropylene/clay nanocomposites. Compos. Sci. Technol..

[16] Medvedev G.A., Caruthers J.M. (2015). Stochastic model prediction of nonlinear creep in glassy polymers. Polymer.

[17] Wang X., Pan Y., Qin Y., Voigt M., Liu X., Zheng G., Chen Q., Schubert D.W., Liu C., Shen C. (2018). Creep and recovery behavior of injection-molded isotactic polypropylene with controllable skin-core structure. Polym. Test..

[18] Wu C., Wu R., Tam L.-H. (2022). The creep behavior of semicrystalline carbon nanotube/polypropylene nanocomposite: A coarse-grained molecular study. Polym. Degrad. Stab..

[19] Jorik S., Lion A., Johlitz M. (2019). Design of the novel tensile creep experimental setup, characterisation and description of the long-term creep performance of polycarbonate. Polym. Test..

[20] Smit W., de Vries H. (1970). Rheological models containing fractional derivatives. Rheol. Acta.

[21] Cai W., Wang Z., Wang F. (2024). Temperature and strain-rate dependent fractional constitutive model for glassy polymers. Chaos, Solitons Fractals.

[22] Cai W., Wang P. (2022). Fractional modeling of temperature-dependent mechanical behaviors for glassy polymers. Int. J. Mech. Sci..

[23] Ribeiro J.G.T., de Castro J.T.P., Meggiolaro M.A. (2021). Modeling concrete and polymer creep using fractional calculus. J. Mater. Res. Technol..

[24] Di Paola M., Pirrotta A., Valenza A. (2011). Visco-elastic behavior through fractional calculus: An easier method for best fitting experimental results. Mech. Mater..

[25] Cai W., Wang P., Zhang Y. (2023). Fractional-order model for temperature-dependent rheological behaviors of polymeric materials. Mech. Adv. Mater. Struct..

[26] Cai W., Liu C., Zhang Y. (2024). Fractional damage model of cyclic behaviors for nano-silver paste. Eur. J. Mech. A/Solids.

[27] Cai W., Wang P., Fan J. (2020). A variable-order fractional model of tensile and shear behaviors for sintered nano-silver paste used in high power electronics. Mech. Mater..

[28] Lorenzo C.F., Hartley T.T. (2002). Variable order and distributed order fractional operators. Nonlinear Dyn..

[29] Brito-Oliveira T.C., Moraes I.C., Pinho S.C., Campanella O.H. (2022). Modeling creep/recovery behavior of cold-set gels using different approaches. Food Hydrocoll..

[30] Yu P., Yao X., Han Q., Zang S., Gu Y. (2014). A visco-elastoplastic constitutive model for large deformation response of polycarbonate over a wide range of strain rates and temperatures. Polymer.

[31] Aniskevich K., Starkova O. (2021). Evaluation of the viscoplastic strain of high-density polyethylene/multiwall carbon nanotube composites using the reaction rate relation. Mech. Compos. Mater..

[32] Duan X., Yuan H., Tang W., He J., Guan X. (2021). A phenomenological primary–secondary–tertiary creep model for polymer-bonded composite materials. Polymers.

[33] Lemaitre J. Evaluation of dissipation and damage in metals submitted to dynamic loading. Proceedings of the International Conference of Mechanical Behavior of Materials.

[34] Li Y., Sun X., Zhang S., Han S. (2022). A fractal crazing constitutive model of glassy polymers considering damage and toughening. Eng. Fract. Mech..

[35] Izer A., Barany T. (2010). Effect of consolidation on the flexural creep behaviour of all-polypropylene composite. Express Polym. Lett..

[36] Tanaka Y., Kashiwabara S., Okuya Y. (2016). Time-temperature superposition in the enthalpy relaxation study of polystyrene. Polym. Eng. Sci..

[37] Di Lorenzo S., Di Paola M., La Mantia F.P., Pirrotta A. (2016). Non-linear viscoelastic behavior of polymer melts interpreted by fractional viscoelastic model. Meccanica.

[38] Kodaira Y., Takano Y., Yonezu A. (2022). Characterization of creep deformation behavior of porous polymer membrane under Small-Punch test. Eng. Fail. Anal..

[39] Zhao X., Li D., Wang L.-J., Wang Y. (2022). Role of gelation temperature in rheological behavior and microstructure of high elastic starch-based emulsion-filled gel. Food Hydrocoll..

[40] Mourad A.-H., Fouad H., Elleithy R. (2009). Impact of some environmental conditions on the tensile, creep-recovery, relaxation, melting and crystallinity behaviour of UHMWPE-GUR 410-medical grade. Mater. Des..

[41] Findley W.N., Davis F.A. (2008). Creep and relaxation of nonlinear viscoelastic materials.

[42] Mei S.Q., Tang G., Yang B., Wang Y.F. (2020). Creep/recovery behavior analysis of wood-plastic composites based on fractional order viscoelastic model. Acta Materiae Compositae Sinica.

[43] Liu X., Zhang S., Xu X.J., Zhang Z., Zhou L., Zhang G. (2013). Study on the creep and recovery behaviors of UHMWPE/CNTs composite fiber. Fibers Polym..

[44] Starkova O., Gagani A.I., Karl C.W., Rocha I.B.C.M., Burlakovs J., Krauklis A.E. (2022). Modelling of environmental ageing of polymers and polymer composites-durability prediction methods. Polymers.

[45] Otsuki Y., Hashimoto K., Kobayashi Y., Nishitsuji S., Matsuno H., Ito H. (2025). Analyzing the tensile creep behavior of different types of polypropylenes using a simple fractional differential viscoelastic model. Polymers.

[46] Guedes R.M. (2006). Lifetime predictions of polymer matrix composites under constant or monotonic load. Compos. Part A: Appl. Sci. Manuf..

[47] Amjadi M., Fatemi A. (2021). Creep behavior and modeling of high-density polyethylene (HDPE). Polym. Test..

[48] Nuñez A.J., Marcovich N.E., Aranguren M.I. (2004). Analysis of the creep behavior of polypropylene-woodflour composites. Polym. Eng. Sci..

[49] Zhang Y.-Y., Sun Z., Li Y.-Q., Huang P., Chen Q., Fu S.-Y. (2021). Tensile creep behavior of short-carbon-fiber reinforced polyetherimide composites. Compos. Part B: Eng..

[50] Yang X., Steck J., Yang J., Wang Y., Suo Z. (2021). Degradable plastics are vulnerable to cracks. Engineering.

